# Mannose enhances anti-tumor effect of PLX4032 in anaplastic thyroid cancer

**DOI:** 10.1530/ERC-24-0209

**Published:** 2025-03-24

**Authors:** Zhuolin Li, Liumei Song, Yuanxing Yang, Yang Zhao, Sharui Ma

**Affiliations:** ^1^ Key Laboratory of Shaanxi Province for Craniofacial Precision Medicine Research, College of Stomatology, Xi’an Jiaotong University, Xi’an, China; ^2^ Clinical Research Center of Shaanxi Province for Dental and Maxillofacial Disease, College of Stomatology, Xi’an Jiaotong University, Xi’an, China; ^3^ Department of Cariology & Endodontics, College of Stomatology, Xi’an Jiaotong University, Xi’an, China; ^4^ Department of Endocrinology, Shaanxi Provincial People’s Hospital, Xi’an, China; ^5^ Department of Dermatology, The First Affiliated Hospital of Xi’an Jiaotong University, Xi’an, China; ^6^ Department of Ultrasound, The Second Affiliated Hospital of Xi’an Jiaotong University, Xi’an, China

**Keywords:** mannose, glycolysis, vemurafenib, PLX4032, anaplastic thyroid cancer, ATC

## Abstract

Anaplastic thyroid cancer represents the most aggressive form of thyroid cancer and harbors BRAF mutations in over 40% of cases. Vemurafenib (PLX4032), a BRAF kinase inhibitor, shows promise in BRAFV600E-positive advanced thyroid cancer but may promote resistance in anaplastic cases. This study investigates whether mannose, known to selectively inhibit thyroid cancer, enhances PLX4032 efficacy. To evaluate whether mannose could enhance the response of anaplastic thyroid cancer cells to vemurafenib, we employed several *in vitro* assays, including MTT, colony formation, flow cytometry, migration and invasion assays. In addition, we performed *in vivo* assays using mouse models with subcutaneous xenografts. Our findings demonstrated that vemurafenib and mannose synergistically inhibit anaplastic thyroid cancer cell proliferation. The combined treatment significantly impeded anaplastic thyroid cancer cell migration and invasion while promoting apoptosis. *In vivo* studies corroborated these observations. The underlying mechanism by which mannose potentiates the antitumor effects of vemurafenib was explored using the Seahorse XFe96 Analyzer to measure glycolysis parameters and Western blotting to assess the expression of associated proteins. Mechanistically, vemurafenib reduced the expression of ZIP10, which in turn decreased the enzyme activity of phosphomannose isomerase. This suppression of ZIP10 enhanced mannose-mediated inhibition of glycolysis and thus its antitumor effect, as confirmed by rescue experiments with ZIP10 overexpression. The resulting decrease in glycolysis led to lower ATP levels, which are essential for the phosphorylation of ERK and AKT. Therefore, the combination of vemurafenib and mannose inhibited the levels of pERK and pAKT, thereby improving the effectiveness of PLX4032 in treating anaplastic thyroid cancer.

## Introduction

Anaplastic thyroid cancer (ATC) is a rare endocrine tumor, constituting only 2% of all types of thyroid cancer cases (Molinaro *et al.* 2017). However, it accounts for 14–39% of thyroid cancer-related mortality due to its high malignancy (Cabanillas *et al.* 2016). Patients with ATC have a poor prognosis, with a median survival time of only 5 months and a 1-year survival rate of 20% (Smallridge & Copland 2010). Conventional treatments, including radiotherapy, chemotherapy and surgery, are often ineffective against ATC (Maniakas *et al.* 2022). Therefore, there is a critical need to develop more effective therapeutic strategies for ATC.

Recent studies have investigated the underlying mechanisms of ATC development. Notably, the BRAFV600E mutation has been identified in over 40% of ATC cases (Pozdeyev *et al.* 2018). In normal thyroid tissue with a wild-type BRAF gene, the gene transcribes into BRAF protein, which is activated by the RAS family. However, when the valine at position 600 mutates to glutamic acid (V600E), the glycine-rich domain loses its hydrophobicity, leading to constitutive self-activation. This mutation results in continuous phosphorylation and stimulation of the MAPK/ERK pathway, which is associated with poor prognosis in ATC (Poulikakos *et al.* 2022). Dabrafenib, a BRAF kinase inhibitor combined with trametinib, has been approved for clinical use in ATC (Bible *et al.* 2021). Despite this, prolonged use of BRAF kinase inhibitors in BRAF-mutated ATC patients can lead to resistance in some cases, necessitating the exploration of alternative therapeutic strategies (Zhang *et al.* 2023). Vemurafenib (PLX4032), another BRAF kinase inhibitor, has demonstrated promising antitumor activity in BRAFV600E-positive papillary thyroid cancer patients who were refractory to radioactive iodine, as reported in a phase 2 clinical trial (Brose *et al.* 2016). Therefore, we aim to explore the potential antitumor effect of PLX4032 in combination with other natural compounds.

Mannose, a glucose isomer found in natural foods, has been reported to be safe for human consumption at high doses without adverse effects (Alton *et al.* 1997). It may serve as a potential therapeutic agent for the treatment of urinary tract infections (McCallin *et al.* 2023), type 2 diabetes (Zhang *et al.* 2017) and obesity (Sharma *et al.* 2018), and may also function as a protective agent during chemotherapy for cancer (Ai *et al.* 2023). In addition, mannose has been shown to selectively inhibit cancer cells, with this selectivity linked to phosphomannose isomerase (PMI) expression (Gonzalez *et al.* 2018). Our recent study demonstrated that mannose selectively inhibits thyroid cancer cells by suppressing glycolysis, depending on PMI enzyme activity and ZIP10 expression rather than PMI expression alone (Ma *et al.* 2021). Since BRAF-mutated thyroid cancer highly correlates with the glycolytic metabolic pathway (Nagarajah *et al.* 2015), suppressing glycolysis may enhance the efficacy of PLX4032 in treating ATC. This suggests the potential of mannose to enhance the effectiveness of PLX4032 in treating thyroid cancer.

In this study, we aim to demonstrate the antitumor effects of the combined treatment with PLX4032 and mannose in ATC, both *in vitro* and *in vivo*. Furthermore, we will investigate the underlying mechanisms by which mannose enhances the antitumor effects of PLX4032 through a series of molecular and biochemical experiments.

## Materials and methods

### Measurement of IC50

We seeded ATC cells 8305C and 8505C (2,000–3,000/well) in 96-well plates. After cell attachment, 20 mM D-mannose was added to the culture medium or not, and then different doses of PLX4032 were added to the culture medium at time points. Next, we assessed cell viability using the MTT assay and then calculated the IC50 value of each cell line.

### Cell proliferation assays

Cells (2,000–3,000/well) were seeded in 96-well plates and then treated with 20 mM D-mannose, 4 μM PLX4032, alone, together or not. At 24, 48 and 72 h after seeding, the culture medium was supplemented with a 10% solution of MTT and thereafter placed in an incubator at a temperature of 37°C for a duration of 15 min. After the incubation period, we measured the optical density at a wavelength of 490 nm utilizing a microplate reader.

### Colony formation assays

Cells (3,000–5,000/well) were seeded on 6-well plates and cultured with medium containing 20 mM D-mannose, 4 μM PLX4032, alone, together or not for 7 days. Next, cells were fixed, washed, stained and counted under an inverted microscope. We defined more than 50 cells as a colony. Each assay was carried out in triplicate.

### Cell apoptosis assays

The assessment of cellular apoptosis was carried out employing the FITC Annexin V Apoptosis Detection Kit (BD Biosciences, USA). Initially, cells were seeded on 6-well plates and cultured with medium containing 20 mM D-mannose, 4 μM PLX4032, alone, together or not for 48 h. Next, cells were harvested, rinsed with cold PBS, and subsequently suspended in 100 μL of binding buffer. Then 5 μL staining solution were added, followed by a 20 min incubation in a light-protected environment. Following incubation, the cells underwent a double wash with PBS, were then sieved through a 200-mesh screen, and subsequently analyzed using flow cytometry.

### Caspase activity assay

Caspase activity was measured using the Caspase-3 Activity Assay Kit (Beyotime, China) in accordance with the manufacturer’s protocol. In brief, cells were seeded on 6-well plates and cultured with medium containing 20 mM D-mannose, 4 μM PLX4032, alone, together or not for 48 h. Then cells were collected, lysed and centrifuged, followed by normalization of total protein concentrations. The lysates were then incubated with the respective substrates at a temperature 37°C for a duration of 90 min. The samples were analyzed for absorbance employing a microplate reader (Thermo Scientific, USA) at a wavelength of 405 nm.

### Transwell migration and invasion assay

To perform the migration test, a total of 1 × 10^5^ cells were placed in each chamber with 200 μL of serum-free media on the top insert (8 μm pore size, Corning, USA). For the lower compartment of a 24-well plate, we introduced 800 μL of medium enriched with 20% serum while supplementing 20 mM D-mannose, 4 μM PLX4032, alone or together. After a 24 h incubation period, migrated cells on the underside of the insert membrane were fixed using 100% methanol for a duration of 10 min, followed by 10 min staining in Crystal Violet Staining Solution (Beyotime). In the invasion assay, we adopted a comparable procedure, utilizing Transwell inserts that had been pre-coated with Matrigel (356234; Corning) and rehydrated, with an extended incubation period of 36 h.

### Cell line-derived xenograft model

Female BALB/c/nu nude mice, aged 4–5 weeks, were procured from the Xi’an Jiaotong University Animal Center. Each mouse received a subcutaneous injection on the right flank with 200 μL of 5 × 10^6^ cells, either subjected to transfection using 8305C and 8505C cells, and randomly divided them into four groups when tumor volume grew to 20–30 mm^3^: vehicle control, mannose, PLX4032 and mannose plus PLX4032; five mice/group. For PLX4032 and mannose plus PLX4032 groups, each mouse was injected with PLX4032 at a dosage of 50 mg per kg of body weight once a day. For the mannose and mannose plus PLX4032 groups, normal water was replaced by 15% mannose for the mannose group. Meanwhile, mice received 20% mannose water by oral gavage (150 μL) four times per week. Tumor volumes and body weights were recorded every 3 days, 1 week after implantation. Tumor volume was determined using the formula: volume = length × (width^2^)/2. Following a period of 15 days, the mice were humanely executed by means of cervical dislocation, and tumor tissues were then gathered for the purpose of weighing. The tumor tissues were embedded and processed for immunohistochemistry (IHC) to detect Ki67 (CST). The Institutional Animal Care and Use Committee at Xi’an Jiaotong University granted consent for all treatments.

### Western blot analysis

Cells were cultured and treated with 20 mM D-mannose, 4 μM PLX4032, alone or together. After cells were washed and lysed, equal amounts of protein lysates were subjected to 10% SDS-PAGE electrophoresis and transferred onto polyvinylidene fluoride membranes (Roche Diagnostics GmbH, Germany). Next, we incubated the membranes with primary antibodies at 4°C overnight as follows: anti-ZIP10 (Novus Biologicals, USA), anti-PMI (Abcam, UK), anti-pERK (CST), anti-tERK (Abcam), anti-pAKT (CST), anti-tAKT (Abcam), anti-HER3 (Abcam) and anti-β-actin (Abcam). After being immunoblotted with corresponding secondary antibodies, immunoblotting signals were collected using the Western Bright ECL detection system (Advansta, USA).

### Measurement of PMI enzyme activity

The cysteine carbazole sulfuric acid method was used to measure enzyme activity of PMI as described previously (Sigdel *et al.* 2015). In brief, cells were cultured and treated with PLX4032 or not. Then these cells were washed and then lysed by three freeze–thaw cycles and an ultrasonic cracker on ice. Next, the reactions were initiated by the addition of equal protein samples into the reaction buffer containing 40 mM Tris–HCl pH 7.4, 6 mM MgCl_2_, 5 mM Na_2_HPO_4_/KH_2_PO_4_ and 20 mM mannose-6-phosphatecarbazole. After a 2 h incubation, the reactions were stopped by adding 1.5% cysteine hydrochloride and 0.12% alcoholic solution of carbazole with concentrated sulfuric acid. After shaking this reaction buffer, the amount of complex formed was estimated spectrophotometrically in a spectrophotometer at 560 nm at room temperature. In parallel, β-actin and PMI were detected by western blot analysis to prove protein amount consistency.

### Seahorse glycolytic stress test

The extracellular acidification rate (ECAR), indicative of glycolytic activity, was measured using the Seahorse XF Glycolysis Stress Test Kit (Seahorse Biosciences, USA). Cells were seeded at a density of 2 × 10^4^ cells/well in Seahorse 96-well microplates. After cell attachment, the medium was supplemented with PLX4032 and mannose individually or in combination for 24 h. Then the medium was replaced with base medium containing 2 mM glutamate and incubated for 1 h. Key metabolic modulators were then introduced sequentially: glucose (10 mM), 1 mM oligomycin and 50 mM 2-deoxy-D-glucose, a glycolytic inhibitor (50 mM), at predetermined time points. ECAR measurements were taken to evaluate glycolytic parameters.

### ATP measurement

For ATP measurement, a commercially available firefly luciferase assay kit (Beyotime Institute of Biotechnology, China) was used. Briefly, cells were incubated with PLX4032 and mannose individually or in combination for 24 h. After a single wash with ice-cold PBS, cells were lysed with the ATP-releasing reagent provided by the kit. Then luciferin substrate and luciferase enzyme were added, and bioluminescence was assessed by a fluorescence spectrophotometer. Then the comparable ATP was divided to the total protein.

### ZIP10 overexpression and rescue experiment

8305C and 8505C cells were cultured to achieve 50% confluence and transfected with different lentiviruses encoding PHBLV-ZIP10 and PHBLV-vector (HanBio Biotechnology Co., Ltd, China) at a multiplicity of infection of 10–100. Following successful ZIP10 overexpression, vector control and ZIP10-overexpressing cells were independently treated with a combination of mannose and PLX4032 for 48 h. Cell viability was then assessed using the MTT assay.

## Results

### Mannose enhanced the anti-tumor effect of PLX4032 in BRAF-mutated ATC cells

**Figure 1:**
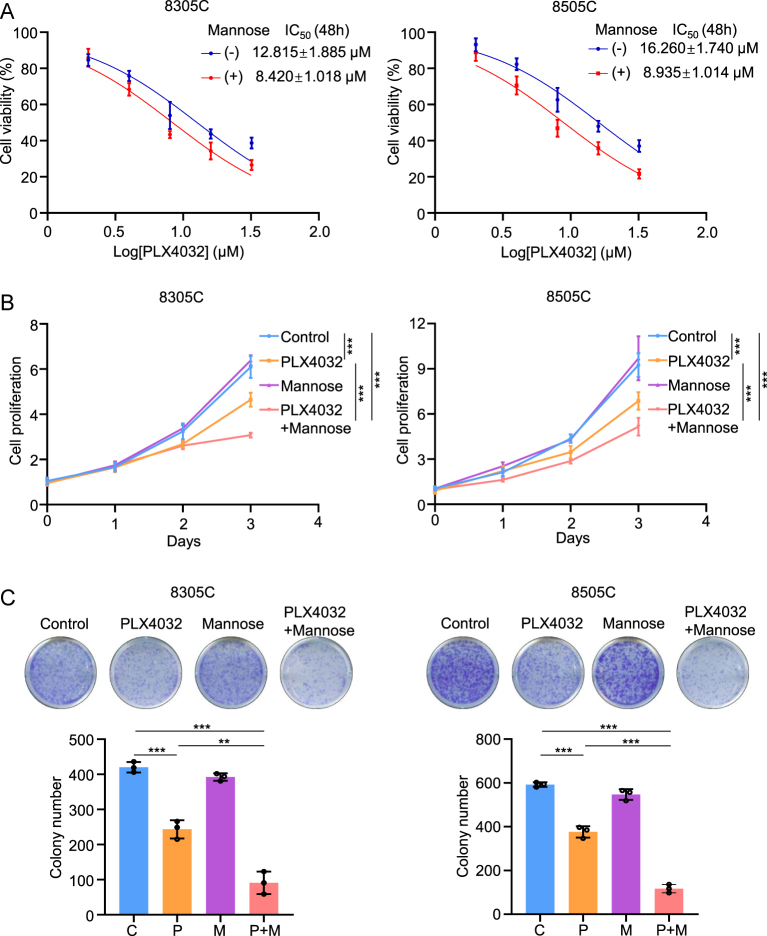
Mannose enhanced the anti-tumor effect of PLX4032 in BRAF-mutated ATC cells. (A) The BRAF-mutated ATC cell lines 8305C and 8505C were treated with PLX4032 in a dose-dependent manner (2, 4, 8, 16 and 32 μM) for 24 h, meanwhile, with or without 20 mM mannose. Cell viability was then evaluated by MTT assay, and IC50 values were calculated using the Reed–Muench method. (B) 8305C and 8505C were treated with 20 mM mannose, 4 μM BRAF kinase inhibitor PLX4032 and the combination of mannose and PLX4032 for 3 days. MTT assays were used to detect cell proliferation. (C) 8305C and 8505C were treated with 20 mM mannose, 4 μM BRAF kinase inhibitor PLX4032 and the combination of mannose and PLX4032 for 7 days. Colony formation assays were used to detect cell proliferation. The data were presented as mean ± SD. Statistically significant differences were indicated: **P < 0.01; ***P < 0.001. A full-color version of this figure is available at https://doi.org/10.1530/ERC-24-0209.

To investigate whether mannose enhanced the anti-tumor effect of PLX4032 in BRAF-mutated ATC, we treated the BRAF-mutated ATC cell lines 8305C and 8505C with increasing doses of PLX4032, both with and without mannose supplementation. MTT assays were performed, and the IC50 was calculated. As shown in Fig. 1A, the IC50 values decreased significantly with mannose supplementation compared to the control (without mannose). Next, we evaluated the proliferation of 8305C and 8505C cells following treatment with a fixed concentration of mannose or PLX4032, alone or in combination, over time. We found that while PLX4032 inhibited cell proliferation, mannose alone did not influence cell proliferation. However, mannose enhanced the cell-inhibition ability of PLX4032 (Fig. 1B). Furthermore, colony formation assays confirmed these results (Fig. 1C). Collectively, our data indicated that mannose improved the anti-tumor effect of PLX4032 in BRAF-mutated thyroid cancer cells.

### Mannose increased PLX4032-induced apoptosis in BRAF-mutated ATC cells

**Figure 2:**
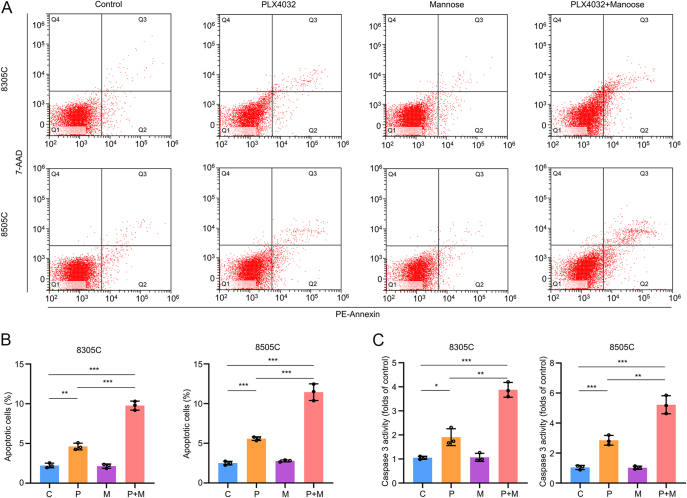
Mannose increased PLX4032-induced apoptosis in BRAF-mutated ATC cells. 8305C and 8505C were treated with 20 mM mannose, 4 μM BRAF kinase inhibitor PLX4032 and the combination of mannose and PLX4032 for 48 h. (A) Representative pictures of apoptosis by flow cytometry. (B) Statistical column of the percentage of apoptotic cells. (C) The activity of caspase 3. Statistically significant differences were indicated: *P < 0.05; **P < 0.01; ***P < 0.001. A full-color version of this figure is available at https://doi.org/10.1530/ERC-24-0209.

In addition to inhibiting cell proliferation, stimulating apoptosis is also a mechanism through which treatments exert their anti-tumor effects. Therefore, we assessed the rate of cell apoptosis in 8305C and 8505C cells following treatment with mannose, PLX4032 or a combination of both. Our data showed that while mannose alone did not influence cell apoptosis, it significantly increased PLX4032-induced apoptosis in BRAF-mutated thyroid cancer cells (Fig. 2A and B). As caspase 3 is crucial for specific processes involved in cellular disassembly and the formation of apoptotic bodies (Porter & Janicke 1999), we also measured caspase 3 activity after the treatments mentioned above. The results were consistent: mannose alone did not affect caspase 3 activity, but when combined with PLX4032, it dramatically increased caspase 3 activity (Fig. 2C). Therefore, we demonstrated that mannose synergized with PLX4032 to induce apoptosis in BRAF-mutated thyroid cancer cells.

### Mannose combined with PLX4032 inhibited ATC cell migration and invasion

**Figure 3:**
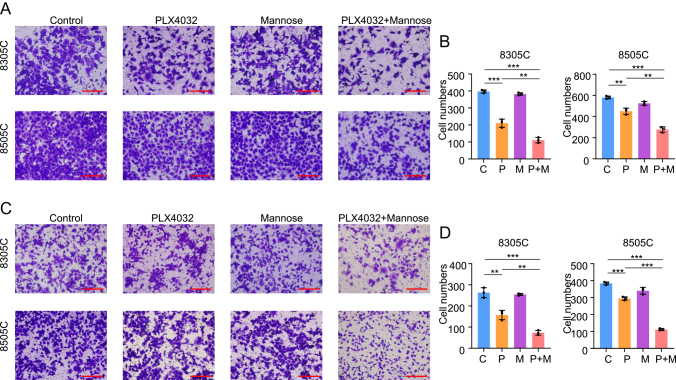
Mannose combined with PLX4032 inhibited ATC cell migration and invasion. 8305C and 8505C were treated with 20 mM mannose, 4 μM PLX4032 and the combination of mannose and PLX4032 for 24 h. (A) Representative pictures of migration transwell assays. (B) Statistical column of migration cell numbers. 8305C and 8505C were treated as mentioned above for 36 h. (C) Representative pictures of invasion transwell assays. (D) Statistical column of invasion cell numbers. Scale bar: 2.0 mm. Statistically significant differences were indicated: **P < 0.01; ***P < 0.001. A full-color version of this figure is available at https://doi.org/10.1530/ERC-24-0209.

A significant difference between cancer cells and normal cells is the ability to migrate and invade, which affects the prognosis of cancer patients. Therefore, we assessed the number of migrated cells in 8305C and 8505C cells following treatment with mannose, PLX4032 or a combination of both. As shown in Fig. 3A and B, mannose alone did not influence migration ability compared to the control, while PLX4032 alone inhibited migration. However, mannose combined with PLX4032 dramatically inhibited cell migration compared to PLX4032 alone. Transwell invasion assays indicated similar results (Fig. 3C and D). Therefore, we demonstrated that mannose combined with PLX4032 inhibited cell migration and invasion in BRAF-mutated ATC.

### Mannose combined with PLX4032 inhibited the progression of ATC *in vivo*

**Figure 4:**
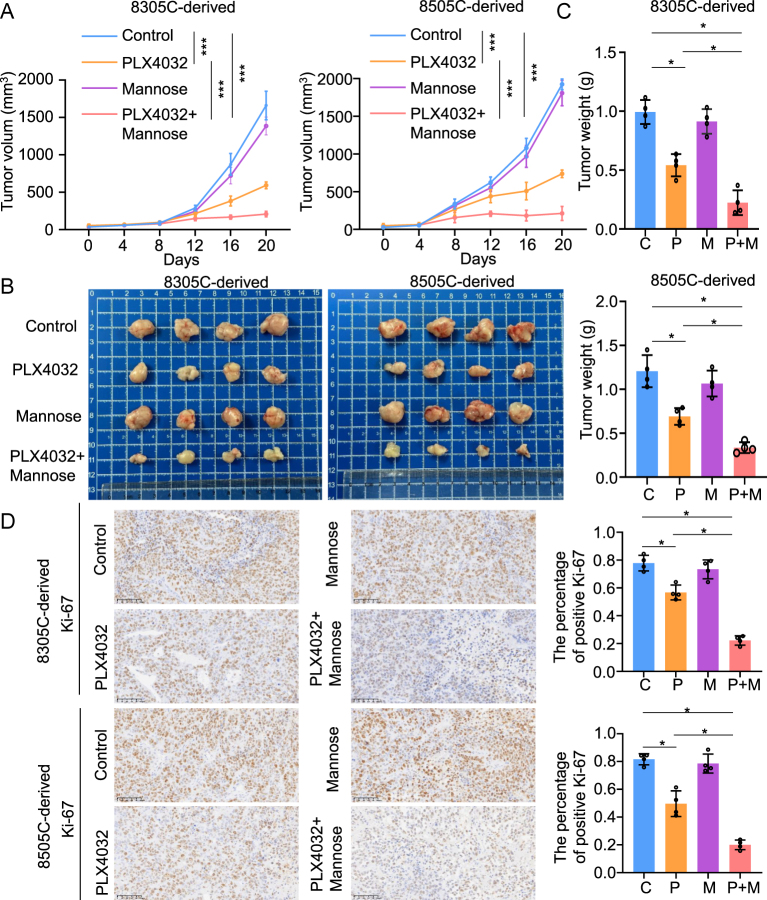
Mannose combined with PLX4032 inhibited progression of ATC *in vivo*. (A) 8305C and 8505C cell-derived xenograft tumors were treated with PLX4032 (50 mg/kg injection), mannose (15% mannose daily water + 20% 150 μL mannose water oral gavage 4/w) or both together. Tumor volume was detected over time. (B) Images of isolated tumors from the above groups after sacrifice. (C) Statistical column of tumor weight of each group. (D) Representative pictures of the expression of Ki-67 of each group by IHC assays on the left panel. The statistical column of positive percentage of Ki-67 on the right panel. Scale bar: 100 μm. Statistically significant differences were indicated: *P < 0.05; ***P < 0.001. A full-color version of this figure is available at https://doi.org/10.1530/ERC-24-0209.

To mimic the *in vivo* environment of thyroid cancer growth, we established a xenograft model using nude mice. We then measured the tumor volume and mouse weight after treatment with mannose, PLX4032 or a combination of both, over time. The results revealed that the combination of mannose and PLX4032 dramatically inhibited tumor burden compared to PLX4032 alone (Fig. 4A). The body weight of the mice showed no significant differences among the treatment groups, including mannose, PLX4032 alone or their combination. In addition, the mice were sacrificed, and the subcutaneous tumors were isolated. The results showed that tumor size and weight decreased significantly following treatment with mannose combined with PLX4032 (Fig. 4B and C). In an *in vivo* environment, the positive percentage of Ki-67 is indicative of the proliferation rate. Therefore, we evaluated the Ki-67 positive index across different treatment groups. The results showed that the Ki-67 positive percentage decreased significantly after treatment with mannose combined with PLX4032 compared to PLX4032 alone (Fig. 4D). Overall, these findings suggested that mannose significantly enhanced the anti-tumor efficacy of PLX4032 in BRAF-mutated ATC.

### Mannose reversed PLX4032 resistance in BRAF-mutant ATC cells by inhibiting reactivation of pERK and pAKT

**Figure 5:**
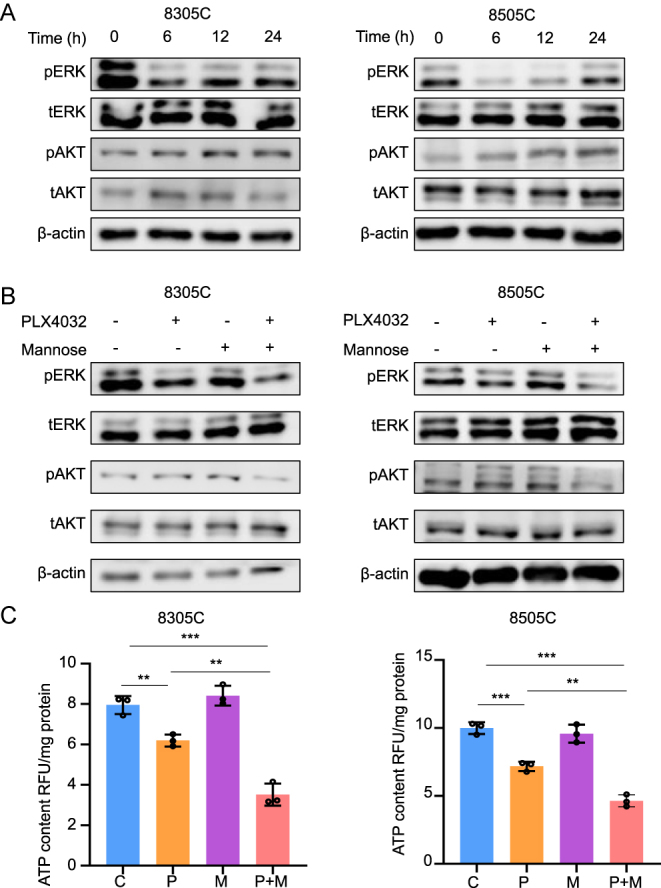
Mannose reversed PLX4032 resistance in BRAF-mutant ATC cells by inhibiting the reactivation of pERK and pAKT. (A) 8305C and 8505C were treated with 4 μM PLX4032 for 6, 12 and 24 h. Western blot analysis of pERK, ERK, pAKT and AKT. β-actin was used as a control. (B) 8305C and 8505C were treated with 20 mM mannose, 4 μM PLX4032 and the combination of mannose and PLX4032 for 24 h. Western blot analysis of pERK, ERK, pAKT and AKT. β-actin was used as a control. (C) 8305C and 8505C were treated with 20 mM mannose, 4 μM PLX4032 and the combination of mannose and PLX4032. ATP Detection Kits were used to test ATP levels. The total amounts of protein were used to normalize ATP levels. Statistically significant differences were indicated: **P < 0.01; ***P < 0.001. A full-color version of this figure is available at https://doi.org/10.1530/ERC-24-0209.

The PI3K/AKT and MAPK/ERK pathways play crucial roles in the progression of thyroid cancer (Scheffel *et al.* 2022). We assessed the protein expression levels of pERK, tERK, pAKT and tAKT following PLX4032 treatment over time. Initially, PLX4032 inhibited pERK and pAKT; however, reactivation occurred at 12 h and persisted (Fig. 5A). Subsequently, we employed a combination therapy of mannose and PLX4032. Notably, mannose alone did not affect pERK, tERK, pAKT or tAKT levels, whereas the combination of mannose and PLX4032 suppressed the reactivation of pERK and pAKT (Fig. 5B). Previous study indicates that PLX4032 reactivates pERK via HER3 activation. To explore this, we assessed mRNA and protein expression of HER3 following treatment with mannose, PLX4032 or their combination. The results showed that PLX4032 upregulated HER3 expression, whereas the combination treatment did not inhibit HER3 levels (Fig. S2). To elucidate the mechanism by which mannose and PLX4032 inhibit pERK and pAKT, we measured ATP levels, an essential phosphate donor and metabolism product (Chu *et al.* 2022). The results indicated that the combination of mannose and PLX4032 significantly reduced ATP levels compared to either treatment alone (Fig. 5C). These findings suggest that mannose reversed PLX4032 resistance in BRAF-mutant ATC cells by inhibiting the reactivation of pERK and pAKT.

### PLX4032 enhanced the glycolysis-suppressive effect of mannose in BRAF-mutant ATC cells

**Figure 6:**
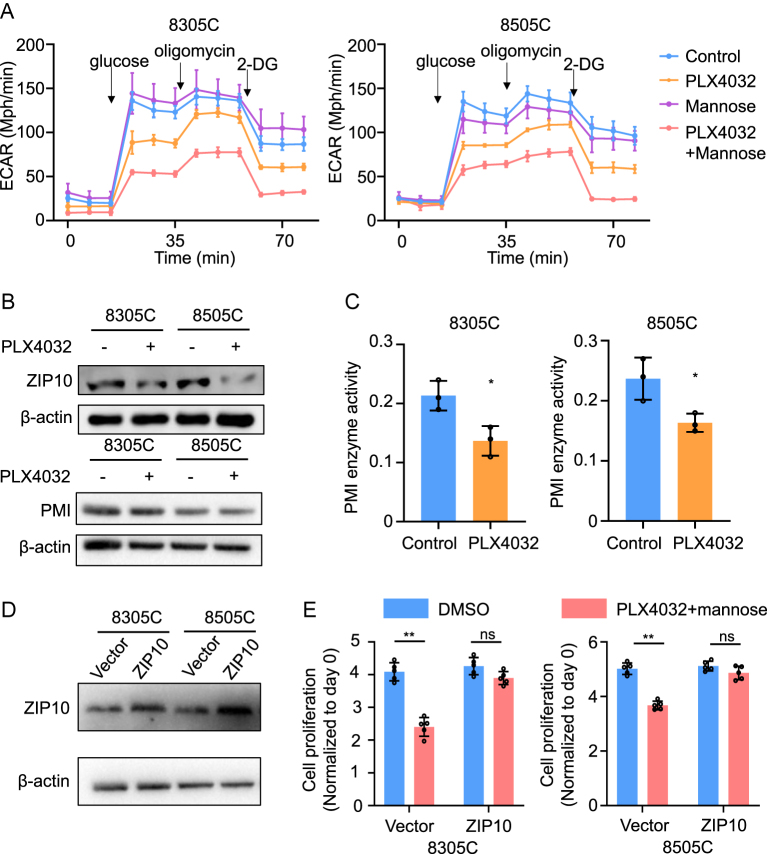
PLX4032 enhanced the glycolysis-suppressive effect of mannose in BRAF-mutant ATC cells. (A) 8305C and 8505C were treated with 20 mM mannose, 4 μM PLX4032 and the combination of mannose and PLX4032 for 24 h. ECAR levels were measured by Seahorse assays after the injection of glucose, oligomycin and 2-deoxy-D-glucose. (B) 8305C and 8505C were treated with 4 μM PLX4032 for 24 h. Western blot analysis of ZIP10 and PMI. β-actin was used as a control. (C) 8305C and 8505C were treated with 4 μM PLX4032 and then PMI enzyme activity was measured. (D) Western blot analysis of ZIP10 after transfection with PHBLV-ZIP10 and PHBLV-vector in 8305C and 8505C cells. β-actin was used as a control. (E) 8305C and 8505C transfected with the indicated lentivirus were treated with 20 mM mannose, 4 μM BRAF kinase inhibitor PLX4032 and the combination of mannose and PLX4032 for 48 h. MTT assays were used to detect cell proliferation (normalized to day 0). Statistically significant differences were indicated: *P < 0.05; **P < 0.01. A full-color version of this figure is available at https://doi.org/10.1530/ERC-24-0209.

ATP is a key product of glycolysis in tumor metabolism due to the Warburg effect (Cornelis *et al.* 2024). Our previous study has shown that mannose selectively kills thyroid cancer by inhibiting glycolysis. We hypothesized that mannose could inhibit glycolysis and consequently reduce ATP levels. Interestingly, Seahorse assays indicated that mannose alone did not significantly alter glycolysis levels compared to the control, while PLX4032 alone partially inhibited glycolysis. Notably, the combination of mannose and PLX4032 led to a significant suppression of glycolysis compared to either agent alone (Fig. 6A). This phenomenon indicated that PLX4032 enhanced the glycolysis-suppressive effect of mannose. Our previous study reported that the glycolysis-suppressive effect of mannose is inversely correlated with ZIP10 expression, which modulates the enzyme activity of phosphomannose isomerase (Ma *et al.* 2021). To determine whether PLX4032 affects ZIP10 and PMI, we analyzed their expression levels and PMI enzyme activity. Western blot assays revealed that PLX4032 treatment decreased ZIP10 expression without altering PMI expression in BRAF-mutant ATC cell lines 8305C and 8505C (Fig. 6B). Furthermore, PLX4032 inhibited PMI enzyme activity (Fig. 6C). These findings suggest that PLX4032 suppresses ZIP10 expression and subsequent PMI activity, thereby enhancing mannose-induced glycolysis suppression in BRAF-mutant ATC cells. To rule out off-target effects of PLX4032, we treated 8305C and 8505C cells with the selective MEK inhibitor GSK1120212, which produced similar results (Fig. S3). To further validate the role of ZIP10, we conducted rescue experiments with ZIP10 overexpression, confirming successful overexpression (Fig. 6D). Overexpression of ZIP10 attenuated the antitumor effects of the combined treatment with PLX4032 and mannose (Fig. 6E). These rescue experiments further confirmed that PLX4032 inhibited ZIP10 expression, thereby enhancing the antitumor effect of mannose.

## Discussion

ATC is the most malignant endocrinologic tumor, associated with a poor prognosis. Approximately 40% of ATC patients harbor BRAF mutations (Pozdeyev *et al.* 2018), for which combined treatment of BRAF kinase inhibitors and MEK inhibitors has been clinically approved (Bible *et al.* 2021). However, their clinical efficacy has been limited (Lang *et al.* 2023). Therefore, there is an urgent need to identify natural ingredients that can enhance the anti-tumor effects of BRAF kinase inhibitors.

Mannose, a safe and naturally occurring sugar, is an isomer of glucose. It utilizes the same transporter as glucose, GLUT1, to enter cells. Once inside, mannose is phosphorylated by hexokinase to produce mannose-6-phosphate, which inhibits glycolysis. However, mannose-6-phosphate can also be converted into fructose-6-phosphate by phosphomannose isomerase (PMI), allowing it to re-enter glycolysis (Sharma *et al.* 2014). In tumor cells with high PMI expression, mannose is readily funneled into glycolysis. Conversely, in tumor cells with low PMI expression, mannose-6-phosphate accumulates and disrupts glycolysis, thereby exerting an anti-tumor effect (Gonzalez *et al.* 2018). Our previous study demonstrated similar findings, showing that mannose selectively kills thyroid cancer cells by inhibiting glycolysis while sparing normal thyroid tissue, even at high doses (Ma *et al.* 2021). In addition, inhibiting glycolysis-related enzymes (e.g., ENO1) has been shown to enhance the efficacy of BRAF kinase inhibitors (Yukimoto *et al.* 2021). In this study, we aimed to evaluate the anti-tumor effects of combined treatment with mannose and PLX4032. Our results demonstrated that mannose and PLX4032 synergistically inhibited the progression of BRAF-mutant ATC, highlighting a potential therapeutic strategy to improve outcomes for these patients.

Previous studies have shown that resistance to BRAF kinase inhibitors in BRAF-mutant thyroid cancer is due to the reactivation of the MAPK/ERK and PI3K/AKT pathwa (Montero *et al.* 2013). Consistent with these findings, our results demonstrated that pERK was initially inhibited by BRAF kinase inhibitor treatment, but both pERK and pAKT were reactivated after prolonged use. Interestingly, while mannose alone did not affect the expression of pERK and pAKT, the combination of mannose and the BRAF kinase inhibitor effectively suppressed the reactivation of both pathways. These findings suggest a synergistic interaction between mannose and the BRAF kinase inhibitor, enhancing their mutual anti-tumor effects.

Our previous study demonstrated that mannose selectively inhibits the progression of thyroid cancer with low expression of ZIP10 and low levels of PMI enzyme activity by suppressing glycolysis. Silencing the expression of ZIP10 reduces zinc influx into cells, which in turn suppresses PMI enzyme activity. When thyroid cancer cells exhibit low ZIP10 and low PMI enzyme activity, mannose-6-phosphate accumulates within cells, leading to glycolysis inhibition. In contrast, high ZIP10 expression and high PMI enzyme activity result in the opposite effect (Ma *et al.* 2021). In this study, we assessed the expression of ZIP10 and PMI enzyme activity after treatment of BARF kinase inhibitor. Interestingly, our results revealed that BRAF kinase inhibitor PLX4032 inhibited ZIP10 expression and subsequently reduced PMI enzyme activity. Moreover, the combination of these two drugs significantly suppressed glycolysis levels, indicating that PLX4032 enhanced the glycolysis-inhibitory effect of mannose. It has been reported that the JAK-STAT-ZIP10-Zn signaling axis influences B-cell homeostasis (Miyai *et al.* 2014). Meanwhile, a recent study demonstrated that ZIP10 is identified as a direct target of c-Myc (Ren *et al.* 2023). Type I mutant-BRAF is the most prevalent mutation in thyroid cancer, occurring at the V600 position (e.g., V600E, V600K, V600D, V600R and V600M). These mutations lead to the expression of RAS-independent, constitutively active monomeric proteins, resulting in robust activation of BRAF activity and sustained activation of the MAPK pathway (Dankner *et al.* 2018). As PLX4032 is a specific inhibitor of type I mutant-BRAF, how type I mutant-BRAF or its downstream MEK regulates ZIP10 needs to be further studied.

BRAF-mutant thyroid cancer cells are known to rely heavily on glycolysis compared to their wild-type counterparts (Nagarajah *et al.* 2015). Suppression of glycolysis has been shown to delay resistance to PLX4032 in melanoma (Brummer *et al.* 2019). Our study similarly demonstrated that inhibiting glycolysis through combined treatment with a BRAF kinase inhibitor and mannose reduces ATP production. ATP is a crucial phosphate provider for the phosphorylation of numerous proteins (Pang *et al.* 2022), resulting in decreased expression of pERK and pAKT after combined treatment with both drugs.

In conclusion, our study indicated that mannose and PLX4032 synergistically inhibited the progression of BRAF-mutant ATC. Specifically, PLX4032 suppressed ZIP10 expression and PMI enzyme activity, thereby facilitating mannose-induced glycolysis inhibition. Given the importance of the glycolysis pathway as an ATP source, the combined treatment effectively reduced ATP levels, thereby reversing PLX4032 resistance by inhibiting the reactivation of pERK and pAKT.

## Declaration of interest

The authors declare that there is no conflict of interest that could be perceived as prejudicing the impartiality of the work reported.

## Funding

This work was supported by the Youth Fund of the National Natural Science Foundation of China (No. 82403902), Shaanxi Province Science Foundation for Youths (No. 2024JC-YBQN-0916 and 2024JC-YBQN-0823), and Doctoral Start-up Fund of Shaanxi Provincial People’s Hospital (No. 2022BS-39).

## Ethics approval and consent to participate

All animal experiments were conducted on the basis of institutional guidelines and were approved by the Laboratory Animal Center of Xi’an Jiaotong University.

## Consent for publication

All authors consent to the publication of this article.

## References

[bib1] Ai YL, Wang WJ, Liu FJ, et al. 2023 Mannose antagonizes GSDME-mediated pyroptosis through AMPK activated by metabolite GlcNAc-6P. Cell Res 33 904–922. (10.1038/s41422-023-00848-6) 37460805 PMC10709431

[bib2] Alton G, Kjaergaard S, Etchison JR, et al. 1997 Oral ingestion of mannose elevates blood mannose levels: a first step toward a potential therapy for carbohydrate-deficient glycoprotein syndrome type I. Biochem Mol Med 60 127–133. (10.1006/bmme.1997.2574) 9169093

[bib3] Bible KC, Kebebew E, Brierley J, et al. 2021 2021 American thyroid association guidelines for management of patients with anaplastic thyroid cancer. Thyroid 31 337–386. (10.1089/thy.2020.0944) 33728999 PMC8349723

[bib4] Brose MS, Cabanillas ME, Cohen EE, et al. 2016 Vemurafenib in patients with BRAF(V600E)-positive metastatic or unresectable papillary thyroid cancer refractory to radioactive iodine: a non-randomised, multicentre, open-label, phase 2 trial. Lancet Oncol 17 1272–1282. (10.1016/s1470-2045(16)30166-8) 27460442 PMC5532535

[bib5] Brummer C, Faerber S, Bruss C, et al. 2019 Metabolic targeting synergizes with MAPK inhibition and delays drug resistance in melanoma. Cancer Lett 442 453–463. (10.1016/j.canlet.2018.11.018) 30481565

[bib6] Cabanillas ME, McFadden DG & Durante C 2016 Thyroid cancer. Lancet 388 2783–2795. (10.1016/S0140-6736(16)30172-6) 27240885

[bib7] Chu XY, Xu YY, Tong XY, et al. 2022 The legend of ATP: from origin of life to precision medicine. Metabolites 12 461. (10.3390/metabo12050461) 35629965 PMC9148104

[bib8] Cornelis JF, Bahar Y, Paola SM, et al. 2024 New insights in ATP synthesis as therapeutic target in cancer and angiogenic ocular diseases. J Histochem Cytochem 72 329–352. (10.1369/00221554241249515) 38733294 PMC11107438

[bib9] Dankner M, Rose AAN, Rajkumar S, et al. 2018 Classifying BRAF alterations in cancer: new rational therapeutic strategies for actionable mutations. Oncogene 37 3183–3199. (10.1038/s41388-018-0171-x) 29540830

[bib10] Gonzalez PS, O’Prey J, Cardaci S, et al. 2018 Mannose impairs tumour growth and enhances chemotherapy. Nature 563 719–723. (10.1038/s41586-018-0729-3) 30464341

[bib11] Lang M, Longerich T & Anamaterou C 2023 Targeted therapy with vemurafenib in BRAF(V600E)-mutated anaplastic thyroid cancer. Thyroid Res 16 5. (10.1186/s13044-023-00147-7) 36855200 PMC9976495

[bib12] Ma S, Wang N, Liu R, et al. 2021 ZIP10 is a negative determinant for anti-tumor effect of mannose in thyroid cancer by activating phosphate mannose isomerase. J Exp Clin Cancer Res 40 387. (10.1186/s13046-021-02195-z) 34886901 PMC8656095

[bib13] Maniakas A, Zafereo M & Cabanillas ME 2022 Anaplastic thyroid cancer: new horizons and challenges. Endocrinol Metab Clin North Am 51 391–401. (10.1016/j.ecl.2021.11.020) 35662448

[bib14] McCallin S, Kessler TM & Leitner L 2023 Management of uncomplicated urinary tract infection in the post-antibiotic era: select non-antibiotic approaches. Clin Microbiol Infect 29 1267–1271. (10.1016/j.cmi.2023.06.001) 37301438

[bib15] Miyai T, Hojyo S, Ikawa T, et al. 2014 Zinc transporter SLC39A10/ZIP10 facilitates antiapoptotic signaling during early B-cell development. Proc Natl Acad Sci U S A 111 11780–11785. (10.1073/pnas.1323549111) 25074913 PMC4136617

[bib16] Molinaro E, Romei C, Biagini A, et al. 2017 Anaplastic thyroid carcinoma: from clinicopathology to genetics and advanced therapies. Nat Rev Endocrinol 13 644–660. (10.1038/nrendo.2017.76) 28707679

[bib17] Montero CC, Ruiz LS, Dominguez JM, et al. 2013 Relief of feedback inhibition of HER3 transcription by RAF and MEK inhibitors attenuates their antitumor effects in BRAF-mutant thyroid carcinomas. Cancer Discov 3 520–533. (10.1158/2159-8290.cd-12-0531) 23365119 PMC3651738

[bib18] Nagarajah J, Ho AL, Tuttle RM, et al. 2015 Correlation of *BRAF*^V600E^ mutation and glucose metabolism in thyroid cancer patients: an ^18^F-FDG PET study. J Nucl Med 56 662–667. (10.2967/jnumed.114.150607) 25814520 PMC4970467

[bib19] Pang K, Wang W, Qin JX, et al. 2022 Role of protein phosphorylation in cell signaling, disease, and the intervention therapy. MedComm 3 e175. (10.1002/mco2.175) 36349142 PMC9632491

[bib20] Porter AG & Janicke RU 1999 Emerging roles of caspase-3 in apoptosis. Cell Death Differ 6 99–104. (10.1038/sj.cdd.4400476) 10200555

[bib21] Poulikakos PI, Sullivan RJ & Yaeger R 2022 Molecular pathways and mechanisms of BRAF in cancer therapy. Clin Cancer Res 28 4618–4628. (10.1158/1078-0432.ccr-21-2138) 35486097 PMC9616966

[bib22] Pozdeyev N, Gay LM, Sokol ES, et al. 2018 Genetic analysis of 779 advanced differentiated and anaplastic thyroid cancers. Clin Cancer Res 24 3059–3068. (10.1158/1078-0432.ccr-18-0373) 29615459 PMC6030480

[bib23] Ren X, Feng C, Wang Y, et al. 2023 SLC39A10 promotes malignant phenotypes of gastric cancer cells by activating the CK2-mediated MAPK/ERK and PI3K/AKT pathways. Exp Mol Med 55 1757–1769. (10.1038/s12276-023-01062-5) 37524874 PMC10474099

[bib24] Scheffel RS, Dora JM & Maia AL 2022 BRAF mutations in thyroid cancer. Curr Opin Oncol 34 9–18. (10.1097/cco.0000000000000797) 34636352

[bib25] Sharma V, Ichikawa M & Freeze HH 2014 Mannose metabolism: more than meets the eye. Biochem Biophys Res Commun 453 220–228. (10.1016/j.bbrc.2014.06.021) 24931670 PMC4252654

[bib26] Sharma V, Smolin J, Nayak J, et al. 2018 Mannose alters gut microbiome, prevents diet-induced obesity, and improves host metabolism. Cell Rep 24 3087–3098. (10.1016/j.celrep.2018.08.064) 30231992 PMC6207501

[bib27] Sigdel S, Singh R, Kim TS, et al. 2015 Characterization of a mannose-6-phosphate isomerase from Bacillus amyloliquefaciens and its application in fructose-6-phosphate production. PLoS One 10 e0131585. (10.1371/journal.pone.0131585) 26171785 PMC4718643

[bib28] Smallridge RC & Copland JA 2010 Anaplastic thyroid carcinoma: pathogenesis and emerging therapies. Clin Oncol 22 486–497. (10.1016/j.clon.2010.03.013) PMC3905320 20418080

[bib29] Yukimoto R, Nishida N, Hata T, et al. 2021 Specific activation of glycolytic enzyme enolase 2 in BRAF V600E-mutated colorectal cancer. Cancer Sci 112 2884–2894. (10.1111/cas.14929) 33934428 PMC8253290

[bib30] Zhang D, Chia C, Jiao X, et al. 2017 D-mannose induces regulatory T cells and suppresses immunopathology. Nat Med 23 1036–1045. (10.1038/nm.4375) 28759052 PMC12180587

[bib31] Zhang L, Feng Q, Wang J, et al. 2023 Molecular basis and targeted therapy in thyroid cancer: progress and opportunities. Biochim Biophys Acta Rev Cancer 1878 188928. (10.1016/j.bbcan.2023.188928) 37257629

